# Factors Associated with Participation and Attrition in a Longitudinal Study of Bacterial Vaginosis in Australian Women Who Have Sex with Women

**DOI:** 10.1371/journal.pone.0113452

**Published:** 2014-11-20

**Authors:** Dana S. Forcey, Sandra M. Walker, Lenka A. Vodstrcil, Christopher K. Fairley, Jade E. Bilardi, Matthew Law, Jane S. Hocking, Katherine A. Fethers, Susan Petersen, Clare Bellhouse, Marcus Y. Chen, Catriona S. Bradshaw

**Affiliations:** 1 Melbourne Sexual Health Centre, Alfred Health, Carlton, Victoria, Australia; 2 Melbourne School of Population and Global Health, The University of Melbourne, Carlton, Victoria, Australia; 3 Central Clinical School, Monash University, The Alfred Centre, Melbourne, Victoria, Australia; 4 The Kirby Institute, The University of New South Wales, Darlinghurst, New South Wales, Australia; University of Washington, United States of America

## Abstract

**Objective:**

A number of social and sexual risk factors for bacterial vaginosis (BV) have been described. It is important to understand whether these factors are associated with non-participation or attrition of participants from longitudinal studies in order to examine potential for recruitment or attrition bias. We describe factors associated with participation and attrition in a 24-month prospective cohort study, investigating incident BV among Australian women who have sex with women.

**Study Design and Setting:**

Participants negative for prevalent BV were offered enrolment in a longitudinal cohort study. Participants self-collected vaginal samples and completed questionnaires 3-monthly to endpoint (BV-positive/BV-negative by 24 months). Factors associated with participation in the cohort study were examined by logistic regression and factors associated with attrition from the cohort were examined by Cox regression.

**Results:**

The cross-sectional study recruited 457 women. 334 BV-negative women were eligible for the cohort and 298 (89%, 95%CI 85, 92) enrolled. Lower educational levels (aOR 2.72, 95%CI 1.09, 6.83), smoking (aOR 2.44, 95%CI 1.13, 5.27), past BV symptoms (aOR 3.42, 95%CI 1.16, 10.10) and prior genital warts (aOR 2.71, 95%CI 1.14, 6.46) were associated with non-participation; a partner co-enrolling increased participation (aOR 3.73, 95%CI 1.43, 9.70). 248 participants (83%, 95%CI 78, 87) were retained to study endpoint (BV-negative at 24 months or BV-positive at any stage). Attrition was associated being <30 yrs (aHR 2.15, 95%CI 1.13, 4.10) and a male partner at enrolment (aHR 6.12, 95%CI 1.99, 18.82).

**Conclusion:**

We achieved high participation and retention levels in a prospective cohort study and report factors influencing participation and retention of participants over a 24-month study period, which will assist in the design and implementation of future cohort studies in sexual health and disease.

## Introduction

Dynamic and complex bacterial communities colonise the vagina, and have been shown to undergo fluctuations in composition and abundance ranging from apparent physiological “healthy” states to those that predispose to a number of sequelae. [1]–[6] Bacterial vaginosis (BV) is an adverse state characterised by depletion of key lactobacillus species (spp.), high bacterial loads and marked species diversity, and has been associated with preterm delivery, low birth-weight babies and increased susceptibility to HIV infection. [7] Though many risk factors for BV have been described, the mechanisms by which these influence the vaginal microbiota are not well understood. [6] Recent works have highlighted the need for longitudinal studies to further elucidate the temporal relationship between behaviours and changes in the vaginal microbiota. [8]–[10]

Longitudinal cohort studies require considerable resources and appropriate strategies to optimise recruitment and retention. Failure to do so risks high levels of attrition, threatening studies' internal and external validity. [11] This can be challenging in longitudinal studies of sexual health, due to requirements for collection of genital samples and intimate behavioural information. A number of social and sexual risk factors for BV have been described and it is important to understand whether these factors are associated with non-participation or attrition of participants from longitudinal studies in order to examine potential for recruitment or attrition bias. In addition, it is important to recruit widely from the community in order to maximise the generalisability of the recruited study population.

The Women on Women's Health (WOW) study investigated factors associated with prevalent and incident BV. Women participated in a cross-sectional study and those negative for BV were offered enrolment in a prospective cohort study involving detailed behavioural questionnaires and self-collected vaginal samples 3-monthly over 24 months. The aims of this paper are to describe the methods and strategies employed to achieve high levels of recruitment from the community and optimise retention, and the factors associated with participation and attrition from the cohort. The findings presented herein can inform the development of future studies that seek to investigate the unique interplay between behaviours, relationships and sexual health and disease.

## Methods

### Participants

The WOW study was a 24-month prospective cohort study of 18–55 year-old Australian women who have sex with women (WSW) that was conducted between March 2010 and September 2013. Women were initially recruited to a cross-sectional study investigating factors associated with prevalent BV. [12] Those found to be BV negative at recruitment were then invited to participate in a prospective cohort study. Women were eligible for the cross-sectional study if they were fluent in English and had a female sexual partner (FSP) in the previous 18 months and ineligible if they were pregnant, post-menopausal or had undergone a sex change. Only those without BV by Nugent method [13] on three consecutive weekly swabs at baseline were eligible and invited to join the cohort study.

### Sampling frame and recruitment method

Highly diverse recruitment strategies were employed to enrol women from a broad range of domains within the community. The all-female recruitment team attended major lesbian, gay, bisexual, transgender and intersex (LGBTI) festivals in five capital cities in different states/territories in Australia; Midsumma (Melbourne), Mardi Gras (Sydney), Pride Festival (Brisbane), Springout (Canberra) and Feast Festival (Adelaide), and hosted stalls where women could register their interest and contact details. Meetings with University-based LGBTI groups were held to discuss the study and advertisements were placed on university notice boards, internet sites, bookshops, nightclubs and cafes that specifically target same-sex attracted women in five Australian states and territories (Victoria, Queensland, New South Wales, South Australia and the Australian Capital Territory). Advertisements listed a toll-free telephone number and study website (www.wowhealth.org.au). Investigators conducted media releases on a LGBTI community radio station and a widely advertised WOW event night was held in an inner-city LGBTI venue in Melbourne. Women were also recruited through a number of health services, specifically Melbourne Sexual Health Centre (MSHC), the largest sexually transmitted infection (STI) clinic in Australia, and a General Practice (GP) in Melbourne specialising in lesbian health.

Research staff determined initial eligibility over the telephone and eligible women were mailed a study pack, consisting of study information and a consent form, a questionnaire, three vaginal swab samples and slides (Copan FLOQSwabs; Interpath Services, Melbourne, Victoria, Australia) and instructions on self-collection of vaginal samples, which have been shown to be comparable to clinician-collected samples for diagnosis of BV and have been used extensively in previous studies. [14], [15] Three baseline vaginal samples were self-collected weekly and returned by reply-paid post for diagnosis by Nugent score (NS). [13] Women completed an electronic or paper-based questionnaire with the final sample. If a woman's FSP was a participant in the WOW study, data were linked if both consented and they were categorised as a ‘co-enrolled couple’.

Participants received their BV results by post and/or password-protected website and received AUD$20 for completing the cross-sectional study. Women diagnosed with BV on any of their three self-collected samples were ineligible for the longitudinal cohort study. The results from this cross-sectional study have been previously reported. [12]

### Follow-up of the cohort study

Women negative for prevalent BV were offered enrolment in a 24-month cohort study. Those who enrolled were asked to return a self-collected vaginal swab and behavioural questionnaire three-monthly until study endpoint – BV diagnosed by the Nugent method or no BV at 24 months. Questionnaires recorded considerable detail regarding sexual behaviours with male and female partners, dates of first sexual contact with new partners and dates of last sexual contact if partnerships ended. Women identified sexual partners by name or code, to ensure consistency of relationships longitudinally and for cross-referencing behaviours between co-enrolled couples to investigate potential sexual transmission of BV. At the start of the cohort study, participants were provided with spare packs and were asked to complete an additional questionnaire and two vaginal swabs (seven days apart) on any occasion that they had a new sexual partner (male or female) or were concerned that they had any interim BV symptoms. SMS reminders were sent to all participants at regular intervals during the study to remind them to collect additional samples if they had symptoms or new partners, and to offer further postal packs. They were also provided with study flyers to give new partners if they felt comfortable, which contained study contact information in the event new partners were interested in enrolling in the study.

Participants could complete questionnaires on paper for return by post or via password-protected website. The website provided information about BV and the study, links to health organisations and a platform to contact staff or notify changes in contact details. Participants were telephoned if the follow-up pack was not returned within two weeks. Research staff made up to seven attempts at contact (SMS, telephone call, email) for that interval; contact was then deferred until the next interval. Women diagnosed with incident BV were informed by study staff and could download a results letter with recommended treatment [16] to take to their physician. The primary study outcome was a diagnosis of incident BV, at which point women were then excluded from further follow-up; women who were BV-negative continued to be followed to 24 months. As follow-up was carried out through the standard Australian postal service, women who moved overseas were withdrawn from follow-up.

Participants were given a toll-free number to WOW staff, ensuring rapid responses to enquiries and contact with sexual health physicians for queries. Women were reimbursed with AUD$20 gift vouchers after completion of each three-month study interval, and with an AUD$40 voucher at study endpoint. A study evaluation was completed by those remaining in the study at 12 months. Samples were stored for future analysis by next-generation sequencing and quantitative PCR.

### Generalisability

To assess the generalisability of the study population, baseline demographics and sexual histories reported in the 298 participants in the WOW cohort were compared to: 1) the 215 women self-identifying as ‘homosexual’ (n = 77) or ‘bisexual’ (n = 138) in the Australian Study of Health and Relationships (ASHR) survey (women, n = 9,578) [17] and 2) the 964 same-sex attracted women responding to the Sydney Women and Sexual Health (SWASH) Survey. [18]

### Statistical analysis

Participants testing negative for BV by NS in the baseline cross-sectional study but who declined enrolment in the cohort study were regarded as ‘declined to enrol’. Attrition was defined as enrolment in the cohort study but failure to complete the study to defined endpoints: 1) diagnosis of incident BV or 2) no incident BV diagnosed by 24 months. Attrition was subdivided into formal withdrawal requests and those lost to follow-up. Where reasons for withdrawal were forthcoming, these are included in results. Data were analysed using STATA version 12. [19] The proportions of women who participated and were retained in the cohort were determined. Frequencies were calculated for demographics and behavioural data and we compared: 1) the WOW cohort to lesbian and bisexual respondents in the ASHR and SWASH surveys to assess generalisability, 2) those declining enrolment to those who enrolled in the WOW cohort to determine factors associated with participation and 3) those who completed the WOW cohort study to those lost to follow-up or withdrawn to determine factors associated with attrition. Differences between groups were explored using χ^2^ or 2-sided Fisher's exact tests. Continuous demographic and behavioural variables were compared to the median. Factors associated with declining cohort enrolment were examined using logistic regression to calculate odds ratios (OR, adjusted aOR) and 95% confidence intervals (95%CI).

The attrition rate per 100 person-years was calculated for selected variables, along with Poisson 95%CIs. Hazard ratios (HR, adjusted aHR) for factors associated with attrition were calculated using Cox regression. Individuals' three-monthly observation periods were represented in the dataset allowing variables such as new sexual partners to be recorded separately for each interval. Women were censored when they: 1) withdrew or were lost to follow up, or 2) when they reached a study outcome (BV positive or BV-negative at 24 months). Multivariable models were built using backwards stepwise logistic or Cox regression, including all variables significant on univariate analysis with subsequent removal of highly correlated variables. Analyses controlled for clustering of co-enrolled couples based on unique identifying numbers for each partnership.

Written informed consent was obtained from all participants, and human experimentation guidelines in accordance with those of the National Health and Medical Research Council of Australia were followed in the conduct of clinical research. This study was approved by The University of Melbourne and The Alfred Hospital ethics committee (251/09).

## Results

### Participation and recruitment

**Figure 1:**
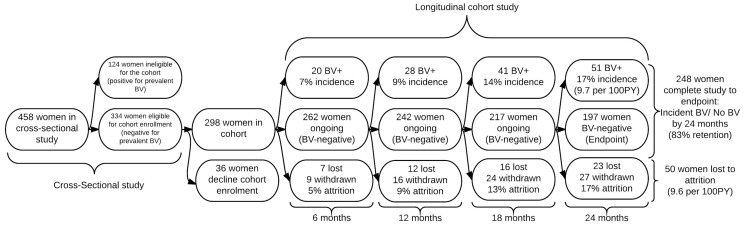
Participation, retention and attrition in the WOW health cohort study.

The majority of women recruited to the WOW cohort heard about the study through advertising at LGBTI festivals (49%), through friends or partners (22%) and the internet (15%). A minority were made aware of the study through posters in universities, cafes and bookshops, and through advertisements on radio and in magazines (10%), or through health services (4%). 458 women were enrolled in the cross-sectional study and BV was detected in 124 women, who were then ineligible to enrol in the cohort study. Factors associated with these prevalent cases have been described previously. [12] Of the remaining 334 women eligible for cohort enrolment (baseline NS 0-6), 298 (89%) completed the minimum requirements for cohort enrolment (≥1 vaginal swab and behavioural questionnaire) and 36 (11%) declined. (***Figure 1***)

### Factors associated with declining to enrol in the cohort study

Most eligible women declining enrolment in the cohort study did not offer a reason; some were ‘too busy’ (n = 4), ‘relocating/moving’ (n = 4) and one had ‘other health issues’. Women co-enrolled with their partner were more likely to enrol in the cohort study (aOR 3.73, 95%CI 1.43, 9.70). Factors associated with declining to enrol in the cohort study by multivariable analysis were not having higher education beyond secondary school (aOR 2.72, 95%CI 1.09, 6.83), smoking (aOR 2.44, 95%CI 1.1, 5.27) and prior genital warts (aOR 2.71, 95%CI 1.14, 6.46). Interestingly, women who reported prior BV symptoms were also less likely to enrol in the cohort (aOR 3.42, 95%CI 1.16, 10.10). *(* ***Table 1***)

**Table 1:** Factors associated with non-participation in the cohort study.

Characteristic	Enrolled in cohort (N = 298) n (%)	Declined cohort enrolment (N = 36) n (%)	Unadjusted odds ratio (95% CI)	*P value* a	Adjusted odds ratio (95% CI)	*P value* a
**Age**						
<30	146 (49)	21 (58)	1.36 (0.66, 2.78)	0.408		
≥30	152 (51)	15 (42)	1			
**Sexual identity**						
Lesbian	218 (73)	29 (81)	1			
Bisexual	60 (20)	7 (19)	0.88 (0.37, 2.06)	0.77		
Other	20 (7)	0 (0)	-	-		
**Country of birth**						
Australia	248 (83)	33 (92)	2.22 (0.65, 7.52)	0.201		
Outside Australia	50 (17)	3 (8)	1			
**Highest educational level**						
Secondary school	40 (13)	10 (28)	2.48 (1.11, 5.53)	**0.026**	2.72 (1.08, 6.83)	**0.033**
>Secondary school	258 (87)	26 (72)	1		1	
**Number of MSPs, last 12 months**						
0	213 (71)	26 (72)	1			
≥1	85 (29)	10 (28)	0.96 (0.44, 2.09)	0.926		
**Number FSPs, last 12 months**						
<1	215 (72)	23 (64)	1			
≥1	83 (28)	13 (36)	1.46 (0.70, 3.05)	0.308		
**Current female sexual partner**						
No	58 (19)	14 (39)	1			
Yes	240 (81)	22 (61)	0.38 (0.18, 0.79)	0.010		
**Co-enrolled with FSP at study onset**						
No FSP/not co-enrolled	156 (52)	30 (83)	1		1	
Yes	142 (48)	6 (17)	0.22 (0.09, 0.55)	0.001	0.27 (0.10, 0.70)	**0.007**
**Current male sexual partner**						
No	288 (97)	33 (92)	1			
Yes	10 (3)	3 (8)	2.62 (0.68, 10.04)	0.160		
**Smoker, last 12 months**						
No	195 (66)	13 (36)	1		1	
Yes	103 (34)	23 (64)	3.35 (1.62, 5.93)	**0.001**	2.44 (1.13, 5.27)	**0.022**
**Past history of BV** b						
No	251 (84)	23 (64)	1		1	
Yes	47 (16)	13 (36)	3.02 (1.42, 6.44)	**0.004**	3.42 (1.58, 10.10)	**0.026**
**Prior genital warts**						
No	281 (94)	30 (83)	1		1	
Yes	17 (6)	6 (17)	3.31 (1.21, 9.00)	**0.019**	2.71 (1.14, 6.46)	**0.024**
***Current vaginal symptoms***						
**Abnormal discharge or odour**						
No	266 (89)	31 (86)	1			
Yes	32 (11)	5 (14)	1.34 (0.50, 3.61)	0.562		

a *P values* controlled for clustering of co-enrolled couples by unique identifier. **Bold text** indicates significant associations at the level **P<0.05.**

b Women in WOW were asked about a history of genital symptoms they thought may have been BV; numbers shown here do not reflect laboratory diagnoses of BV.

### Cohort generalisability

The WOW cohort comprised 298 BV-negative WSW from the cross-sectional study: 1) 122 women co-enrolled with their partner (61 couples) and 2) 176 women enrolled alone. The median age was 30 years, participants predominantly identified as lesbian (73%) or bisexual (20%), and the majority (87%) had higher tertiary education. Most (79%) had a regular female partner, and vaginal sex toy use, receptive digital-vaginal and oral sex were the most common sexual practices. A small number (3%) had a regular partner that was male. Over a third of women (35%) reported smoking in the last 12 months. Prior BV symptoms had been experienced by 16%. (***Table 1*** ***,*** ***2***)

**Table 2:** Baseline demographics, behaviours and longitudinal factors associated with cohort attrition.

Characteristic	Attrition numbers (N = 50) n (%)	Attrition rate per 100 person-years (95% CI)	Unadjusted HR (95% CI)	*P value* a	Adjusted HR (95% CI)	*P value* a
***Baseline variables***						
***Demographics, conditions***						
**Age**						
≤30 years	34 (21.3)	13 (9–19)	2.19 (1.17–4.09)	**0.014**	2.15 (0.13–4.10)	**0.02**
>30 years	16 (11.6)	6 (4–10)	1		1	
**Sexual identity**						
Lesbian	34 (15.6)	9 (6–12)	1			
Bisexual	14 (23.3)	15 (9–25)	1.69 (0.90–3.16)	0.097		
Other	2 (10.0)	6 (1–23)	0.68 (0.09–5.15)	0.706		
**Country of birth**						
Australia	43 (17.3)	8 (4–17)	1.18 (0.53–2.65)	0.684		
Outside Australia	7 (14.0)	10 (7–13)	1			
**Highest educational level**						
Secondary school	8 (20.0)	12 (6–25)	1.34 (0.62–2.89)	0.462		
Higher education	42 (16.3)	9 (7–12)	1			
**Past history of BV** b						
No	40 (15.9)	9 (6–12)	1			
Yes	10 (21.3)	14 (8–26)	1.66 (0.77–0.59)	0.195		
***Baseline sexual behaviours***						
**Number of FSPs, last 12 months**						
≤1	28 (13.0)	7 (5–10)	1		1	
>1	22 (26.5)	18 (12–27)	2.71 (1.49–4.94)	**0.001**	1.90 (0.98–3.68)	0.059
**Number of MSPs, last 12 months**						
0	28 (15.5)	9 (6–13)	1			
≥1	22 (18.8)	11 (7–17)	1.26 (0.70–2.27)	0.446		
**Current male sexual partner**						
No	45 (15.6)	9 (7–12)	1		1	
Yes	5 (50.0)	55 (23–131)	7.12 (2.97–14.05)	**<0.001**	6.12 (1.99–18.82)	**0.002**
**Current female sexual partner**						
No	12 (19.3)	12 (7–21)	1			
Yes	38 (16.1)	9 (7–12)	0.74 (0.38–1.44)	0.379		
***Longitudinal variables*** c						
**Smoking**						
No	27 (13.1)	7 (5–11)	1		1	
Yes	23 (25.0)	15 (10–23)	2.15 (1.24–3.73)	**0.007**	1.65 (0.91–6.01)	0.099
**Couple co-enrolled**						
No	35 (17.7)	11 (8–15)	1			
Yes	15 (15.0)	7 (4–12)	0.70 (0.35–1.42)	0.323		
**New male sexual partner**						
No	44 (15.4)	9 (7–12)	1			
Yes	6 (46.2)	27 (12–60)	3.11 (1.28–7.61)	**0.013**		
**New female sexual partner**						
No	42 (16.6)	9 (7–12)	1			
Yes	8 (17.8)	18 (9–35)	1.59 (0.68–3.74)	0.285		
***Current vaginal symptoms***						
**Abnormal discharge or odour**						
No	45 (16.4)	9 (7–12)	1			
Yes	5 (20.8)	16 (6–37)	1.80 (0.73–4.43)	0.199		
***BV History in partners***						
**Do you think any FSPs have had BV in the past**						
No	37(17.4)	10 (7–13)	1			
Yes	3 (10.7)	7 (2–21)	0.72 (0.22–5.30)	0.575		
Don't know	10 (15.9)	11 (6–21)	1.22 (0.61–2.45)	0.577		

a *P values* controlled for clustering of co-enrolled couples by unique identifier. **Bold text** indicates significant associations at the level *P<0.05*.

b Women in WOW were asked about a history of genital symptoms they thought may have been BV; numbers shown do not reflect laboratory diagnoses of BV.

c Each variable is comprised of behaviours reported longitudinally by participants at each study interval. For raw data, numbers reflect presence or absence of the exposure in the interval preceding participants' attrition.

Limited published data are available that provide sufficient detail about sexual behaviours in WSW with which to compare our cohort. We compared the WOW cohort to the only available Australian community-based data sets for WSW: the Australian Study of Health and Relationships (ASHR) survey [17] and the Sydney women and Sexual Health (SWASH) survey. [18] ASHR surveyed 9,578 women nation-wide over the telephone in a study of relationships and sexual health in Australia. Most responses were only stratified according to gender, however limited demographic and sexual behaviour data were presented in the survey for 215 women self-identifying as ‘homosexual’ (n = 77) and ‘bisexual’ (n = 138). [17] Compared to the these participants, those in the WOW cohort had higher levels of education, were more likely to identify as lesbian and were more likely to have had a female partner and less likely to have had a male partner in the last 12 months. [17], [20] The SWASH survey, conducted biennially at Mardi Gras in Sydney involved a larger LGBTI sample (n = 964) [18] and the demographics and behavioural characteristics in SWASH showed considerable similarity to the WOW cohort. Both WOW and SWASH studies contained higher proportions of lesbians than bisexual women, thereby differing from the ASHR survey participants, and sexual behaviours did not differ markedly between the WOW and SWASH participants. WOW participants however, had higher levels of education and more likely to be in a current relationship with a woman than participants in the SWASH survey.

### Retention and attrition from the cohort study – proportions

**Figure 2:**
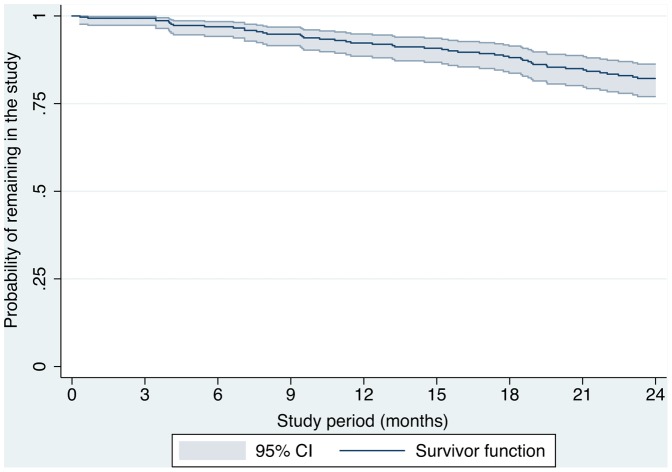
Time under observation until loss to follow-up or withdrawal.

Of the 298 (89%, 95%CI 85, 92) women returning the minimum requirements for longitudinal cohort enrolment, 248 (83%, 95%CI 78, 87) completed the cohort study to endpoint (incident BV or no BV by 24 months). The number of participants who developed incident BV, were retained and BV-negative or were lost to follow-up or withdrew at each six month interval to endpoint is shown in ***Figure 1***. Of the 248 retained participants, 172 (69%) handed in every study pack until their endpoint; 76 (31%) participants skipped ≥1 intervals of follow-up. New partner or symptom packs (n = 64) were utilised by 38 (13%) individual women between study intervals. 27 withdrew and 23 were lost to follow-up, yielding an attrition rate of 9.6 per 100PY (95%CI 7, 12). Reasons for withdrawal included ‘moving/relocating’ (n = 10), ‘too busy’ (n = 4), ‘health issues/pregnancy’ (n = 3) or ‘new boyfriend’ (n = 1); 9 women did not offer a reason. ***Figure 2*** displays time under observation until either withdrawal, loss to follow-up, incident BV or no incident BV by 24 months. The number of participants successfully completing each stage of the study was 282 (95%) at 6 months, 270 (91%) at 12 months, 258 (87%) at 18 months and 248 (83%) at 24 months.

### Predictors of retention and attrition in the cohort study

To investigate factors associated with attrition, participants completing the study to defined endpoints (n = 248) were compared to those lost before endpoint (n = 50). (***Table 2***) No specific sexual behaviours with FSPs were significantly associated with attrition by univariate analysis. A number of sexual behaviours with male sexual partners (MSPs) were significantly associated with attrition by univariate analysis, but these and bisexual identity was highly correlated with reporting a new MSP and reporting a current MSP at baseline. Reporting a current MSP at enrolment was most strongly associated with attrition, and was therefore retained in the multivariate model. In adjusted analyses, attrition was associated with report of a current MSP at enrolment (aHR 6.12, 95%CI 1.99, 18.82) and younger age (<30 years) (aHR 2.15, 95%CI 1.13, 4.10), but not with smoking.

### Study evaluation

At 12 months, 258 surveys were posted to participants remaining in the study, which were completed by 189 (73%, 95%CI 67, 78) retained women. (***Table 3***) Women reported a high degree of satisfaction with the study – 88% felt ‘good’ about the study and 99% found study packs easy to understand and samples easy to collect. Although the majority (71%) had never heard of BV at baseline, most (81%) knew more about the condition by 12 months and thought BV testing was a good idea (98%). A high proportion (84%) passed information about the study onto other women. Key motivating factors for participation included ‘research in general’ (74%) and ‘interest in women's/lesbian health issues’ (69%). Only half (54%) cited reimbursement vouchers as an incentive to join the study; some noted that the vouchers were appreciated but were not a motivating factor for joining the study. Interestingly, despite the high uptake of technology in the community, only half (57%) used the study website and 84% preferred paper questionnaires. There were no significant associations between responses to the 12-month study evaluation and attrition (p≥0.093).

**Table 3:** 12-month study evaluation responses from 189 respondents.

Questions	Number of respondents (N = 189) n (% {95% CI})
**How do you feel about the study?**	
Somewhat awkward/very awkward	23 (12.3 {8.2, 18.3})
Good	162 (87.6 {81.7, 91.8})
**Did you find the WOW pack easy to understand?**	
No	2 (1.1 {0.2, 4.2})
Yes	184 (98.9 {95.8, 99.8})
**How do you find self-collecting samples?**	
Not at all easy	2 (1.1, {0.2, 4.2})
Very easy/somewhat easy	184 (98.9 {95.8, 99.8})
**How much did you know about BV before the study?**	
Never heard of it	134 (71.3 {61.1, 77.5})
Knew a little/Knew a lot	54 (28.7 {22.5, 35.8})
**Do you think you know more about BV now than at the start of the study?**	
No	36 (19.3 {14.0, 25.8})
Yes	151 (80.8 {74.2, 86.0})
**Did you pass information about WOW onto anybody?**	
No	31 (16.5 {11.6, 22.8})
Yes	157 (83.5 {74.2, 86.0})
**Do you think testing for BV is a good idea?**	
No	31 (16.5 {11.6, 22.8})
Yes	157 (83.5 {74.2, 86.0})
**What were your motivations for joining the WOW study?£**	
Research in general	139 (73.5 {66.6, 79.6})
Interest in women's health/lesbian health issues	131 (69.3 {62.1, 75.7})
Vouchers	102 (54.0 {46.6, 61.2})
What are your preferred methods of contact for the WOW study?^d^	
Email	105 (55.6 {48.2, 62.7})
SMS	37 (19.6 {14.3, 26.1})
Phone Call	29 (15.3 {10.7, 21.5})
Post	11 (5.8 {3.1, 10.5})
Any option/Don't mind	30 (15.9 {11.1, 22.1})
**What is your preferred method of completing study questionnaires?**	
Online	24 (12.9 {8.6, 18.8})
Paper	159 (85.5 {79.4, 90.1})
Either	3 (1.6 {0.4, 5.0})
**Did you find the WOW website helpful?**	
Yes	75 (39.9 {32.9, 47.3})
No	3 (1.6 {0.4, 5.0})
Did not use	110 (58.5 {51.1, 65.6})

d May add up to>100% as participants could pick ≥1 response

## Discussion

The WOW cohort study is one of the largest prospective cohort studies to date investigating the influences of behaviours and relationship characteristics on the vaginal microbiome and incident BV. Women were recruited from diverse settings and we report high levels of participation and retention over a 24-month study period. We identified a number of important characteristics that influenced a woman's decision to join the WOW cohort study and remain in it. Women were less likely to enrol in the prospective cohort if they smoked, had lower educational levels, or a history of BV symptoms or of genital warts, and enrolment was strongly associated with a partner co-enrolling in the cohort. Attrition was associated with reporting a current male partner at baseline and being less than 30 years of age. Because some of these characteristics have been associated with BV in past studies, understanding how these factors influence cohort participation and retention is important to minimise bias and assist in the future design of studies and their interpretation. A mid-study evaluation investigated women's motivations for participating in the study and demonstrated very high acceptability of study procedures and process, significant increases in BV knowledge and high rates of study referral to others. Understanding motivations for participating in studies and satisfaction with study methodology and involvement is also integral to improving the future design of successful prospective cohort studies.

The vaginal microbiota is a dynamic environment influenced by a range of hormonal and immunological factors and human behaviours, [21], raising questions about the capacity of cross-sectional studies to associate composition of the vaginal microbiota with health outcomes. [6] Cohort studies not only enable investigation of events antecedent to disease diagnosis but also inform strategies to identify those at risk and develop targets for prevention and intervention. [21] It is challenging to maximise retention and minimise loss to follow-up bias in longitudinal studies. These challenges are compounded when the research involves sensitive subject matter, such as sexual health issues, and when studies require frequent self-collection of samples during follow-up.

To maximise community recruitment, the WOW study successfully employed diverse methods to enhance recruitment and community-based drives recruited 49% of participants. Social media and internet-based advertising recruited 15% of participants. Women were encouraged to inform others about the study and 22% of participants reported hearing about the study through their social circles and 84% reported that they passed the study information onto others. Women co-enrolled in the cross-sectional study with a FSP were more likely to enrol in the cohort study, indicating that enrolment of couples may be a successful recruitment strategy. Although this strategy may be a source of bias for some studies, for our study this feature facilitated the study of potential transmission of BV between couples.

WSW are under-represented in medical and sexual health research. [22] Very limited published data was available against which to assess the generalisability of the WOW cohort. We compared the WOW cohort to two Australian community-based studies, respondents to the SWASH survey [18] and the self-identifying ‘homosexual’ and ‘bisexual’ respondents in the larger nationwide ASHR survey. [17] The proportions of women identifying as WSW in the ASHR study were low, [17] and this population may have been under-represented. The SWASH survey may have experienced selection bias towards festivalgoers, and may not necessarily be representative of the broader WSW community. While the WOW study may have self-selected for women interested in participating in research, however efforts were made to ensure recruitment from a diverse range of community venues nationwide. Both the ASHR [17] and SWASH surveys [18] sampled participants less educated than the WOW cohort, which was not due to sampling bias of younger women. As we have demonstrated that less educated women were less likely to commit to a longitudinal study, it is unsurprising that our cohort were more educated than those completing a once-off surveys. In contrast to the ASHR survey, the WOW cohort had a higher proportion of women identifying as lesbian than bisexual but importantly the WOW cohort was similar to SWASH participants in this respect and sexual practices among the WOW cohort were not markedly different from those reported in SWASH. [18] The WOW cohort was therefore broadly comparable to other community-based studies of self-identifying WSW and importantly may be more generalisable than other published BV studies that recruit predominantly from medical and STI clinics [23]–[28] as low rates of healthcare attendance are reported in this population. [18]

The participation characteristics associated with enrolment, such as women enrolling with their partner being more likely to enrol and those with a lower education level, smokers and history of genital warts and past BV symptoms less likely to enrol indicates potential enrolment of women with a lower STI/BV risk profile. Past BV symptoms may be a marker of unmeasured risk, but importantly, women reporting past BV symptoms did not have higher numbers of recent or lifetime partners or differences in sexual behaviours than those without a history of BV symptoms. Participants with lower levels of education are less likely to participate and remain in longitudinal studies, [29]–[32] so our findings are not unexpected but do highlight the need to develop strategies to engage these participants in research and cohort studies. Once enrolled in the cohort study, reporting a male partner at baseline and being younger than 30 years were associated with attrition. Future studies among this population may consider whether exclusion of women with a current MSP improves participant retention in studies of WSW, though this would need to be balanced against the risk of creating a sampling bias among the population recruited for investigation. This should clearly not be extended to exclusion of women with any history of male sexual partners as the majority of WSW report a lifetime history of sexual contact with males. [33], [34] Younger age has been shown in antecedent studies to be associated with higher attrition rates and again highlights the need for novel strategies to retain younger people in prospective studies. [29], [30] Although smokers were less likely to enrol in the cohort, these numbers were small and overall smoking was not associated with attrition. Some studies have linked smoking and BV [12], [26], [35] and it is possible that having fewer smokers enrolled reduced the risk of incident BV in the cohort and limited our ability to examine the association between smoking and BV.

Participant retention is essential for a quality cohort study, as attrition can impact studies’ power, internal and external validity. [30] Successful tools to improve retention in longitudinal studies include reimbursements, reminders, self-sampling options and providing alternative methods of data collection, [9], [36], [37] many of which were included in WOW and resulted in the high proportion retained. Preferred method of contact during the study varied, highlighting the importance of providing multiple avenues of communication available to participants. [37] Studies requiring regular examination by clinicians are associated with increased loss to follow-up, [9] and as 99% of WOW participants found samples easy to self-collect, allowing sample self-collection may improve retention in longitudinal studies. Future studies should therefore aim to utilize and offer highly diverse recruitment and retention strategies to maximize engagement of younger, less educated, at risk participants. While we had a website and Facebook page and advertised on a number of online gay sites and gay media, rapid advances in technology and the increasing use of social media creates exciting opportunities for study information to be even broadly disseminated in the community. Although automated text messaging reminders are an effective method to improve retention and relevant for young participants, development of other tools including smartphone “apps” with calendar functions can facilitate engagement and generate reminders. Women in our study cited an interest in women's and lesbian health and research in general for participating in the study, and importantly, females' participation in research has been shown to be enhanced when they are aware that their participation is important and will benefit others and themselves. [31] Educating younger and less educated women of the benefits that research may offer their peer-group may also be an important strategy to promote participation and retention.

Our study has a number of limitations to consider. Studies with long periods of follow-up need to consider sampling frequency and duration so it is not unduly onerous for participants, but the WOW study's relatively long interval periods may have resulted in failure to detect BV between intervals and risks recall bias for behavioural data. To counter these potential problems, we encouraged women to complete additional samples and questionnaires if they were concerned about symptoms or had a new sexual partner, which 13% did at some stage during the study. Our study population was more highly educated compared to those in other studies of WSW [17], [18] and predominantly Australian-born and therefore may not be generalisable to the wider WSW population. However, sexual behaviours did not differ markedly from other studies of WSW. [18] We demonstrated recruitment bias of more educated women and non-smokers and attrition of women <30 years and those reporting a male partner at baseline. A potential source of attrition bias in this study is that, in keeping with standard analytical methodology, women who were lost to follow-up were compared to women who reached the study endpoint, either incident BV or no incident BV by 24 months. As the former group contributes less observational time to attrition analyses, and the epidemiological characteristics of women with BV are known to differ from those without (i.e. BV is associated with higher numbers of sexual partners and inconsistent condom use), [38] this analysis is potentially biased towards identification of risk factors for attrition that are more common in women without BV. Half of our cohort was recruited from festivals, in contrast to the majority of published BV studies that recruit from medical services. [23]–[28] In addition, a large proportion of women were recruited by referral from others enrolled in the WOW study. Snowball sampling may impart additional biases, [39], [40] but also has advantages as community recruitment can help to recruit populations often overlooked and disconnected from medical services [18], [22] and has a strong history of affirmative research in LGBTI communities. [39]

### Conclusions

Variations in the vaginal microbiota limit the capacity for cross-sectional studies to fully characterise the temporal relationship between risk factors and BV acquisition however cohort studies are challenging due to lengthy follow-up periods requiring collection of intimate samples and sensitive behavioural information. These factors can conspire to make recruitment difficult and risks participant attrition, threatening studies' internal and external validity. Despite these challenges, the WOW study methodology achieved high proportions of recruitment from diverse community settings and retained a high proportion of participants. Study evaluation gave insight into motivations for participation and views on the study experience, about which women were overwhelmingly positive. Further work needs to be done in order to access and recruit less educated women and retain younger women in studies. It is hoped that our findings may inform future cohort studies of sexual health and disease.
